# Study of cytomorphology of solid pseudopapillary tumor of pancreas and its differential diagnosis

**DOI:** 10.4103/0970-9371.73293

**Published:** 2010-10

**Authors:** Neelam Mehta, Lopa Modi, Trupti Patel, Manoj Shah

**Affiliations:** The Gujarat Cancer and Research Institute, Ahmedabad, Gujarat, India

**Keywords:** Solid pseudopapillary tumor, pancreas; aspiration cytology, ultrasound guided

## Abstract

**Background:**

Solid pseudopapillary tumor is a rare pancreatic neoplasm with uncertain to low malignant potential. This is an uncommon neoplasm with many pseudonyms, occurring predominantly in young woman under the age of thirty years.

**Aims:**

To study the cytomorphological features of six cases of solid and pseudopapillary epithelial neoplasm of pancreas diagnosed on fine needle aspiration cytology (FNAC) in years 2005 to 2007 and its cyto-histological correlation.

**Materials and Methods:**

Image-guided FNAs was done in these six patients preoperatively. Alcohol-fixed smears were stained with Papanicolaou stain, cytomorphological findings were evaluated and diagnosis was made. Diagnosis was later confirmed by histology in all cases.

**Results:**

All six cases show characteristic cytological features such as hypercellular smears with presence of abundant delicate papillary fragments, dyscohesive cells, monomorphic tumor cells with delicate folded nuclear membranes, and foamy macrophages in the background.

**Conclusions:**

Preoperative correct diagnosis of solid pseudopapillary tumor of pancreas is possible on FNAC and by doing so it helps in management of this surgically curable neoplasm.

## Introduction

Solid pseudopapillary tumor (SPT) is the most recent descriptive term of this characteristic; however, enigmatic pancreatic tumor has previously been designated by many pseudonyms such as “solid and papillary tumor”, “papillary cystic tumor”, “solid and cystic tumor”, “solid cystic and papillary epithelial neoplasm”.[1] Since degenerating cystic change is characteristic of SPT, SPT must be considered in differential diagnosis of pancreatic cystic neoplasm and with pancreatic pseudocyst.

Solid pseudopapillary tumor is a distinctive and rare tumor of the pancreas having low malignant potential and is seen predominantly in adolescent girls and young women but cases from men are also reported.[2–5] An accurate preoperative diagnosis is highly desirable since these patients can have long survival with adequate surgery.[6] The cytological features of this tumor are highly characteristic and it is possible to differentiate it from other pancreatic tumors with relative ease.[6]

Here we have described six cases of SPT of the pancreas diagnosed preoperatively on fine needle aspiration cytology (FNAC).

## Materials and Methods

Ultrasound-guided percutaneous FNAs were performed preoperatively using 22G needle attached to 10 ml plastic syringe by interventional radiologist in our institute. Smears were made on glass slides, fixed immediately in 95% alcohol for subsequent Papanicolaou staining. Additional aspiration material was used for the cell block preparation. Cytomorphological evaluation of smears was done for cellularity, cell type, nuclear details, and background and cytologic diagnosis were made which was later on confirmed by histopathology. Immunohistochemistry studies were performed on cell block material using polymer-linked technique (Novocastra; UK) using antibodies against cytokeratin (dilution 1:50), epithelial membrane antigen (dilution 1:50), vimentin (dilution 1:20), synaptophysin (dilution 1:50), neurone-specific enolase (dilution 1:50) and CD-56 (dilution 1:50).

## Results

Between the years 2005 and 2007, six female patients age ranging from 16 to 35 years presented at our institute with symptoms related to a mass in the abdomen. Clinical and ultrasonography finding of all the six patients are summarized in Table 1.

**Table 1:** Clinical and USG findings

Case	Age/Sex	Clinical finding	Tumor location	USG findings	Size mm	Type of surgery	Histology diagnosis
1.	16 yrs/F	Pain in abdomen×1 month O/E Lump in abdomen	Head	Mixed echogenic well circumscribed	80×65	Biopsy (inoperable)	SPEN
2.	35 yrs/F	Dyspepsia×2 months	Head	Mixed echogenic well circumscribed	67×61	Duodenum sparing pancreatectomy	SPEN
3.	23 yrs/F	Pain in abdomen O/E lump in abdomen	Tail	Mixed echogenic well circumscribed	100×120	Excision of mass	SPEN
4.	20 yrs/F	Lump in abdomen×1½ yrs	Body and tail	Mixed echogenic well circumscribed	160×140	Total pancreatectomy	SPEN
5.	20 yrs/F	Pain in abdomen×2 yrs, vomiting	Head and body	Mixed echogenic well circumscribed	97×64	Biopsy	SPEN
6.	30 yrs/F	Lump in abdomen×1 month	Head, uncinate process and extending to body	Mixed echogenic well circumscribed	80×70	Enucleation of tumor	SPEN

**Figure 1:**
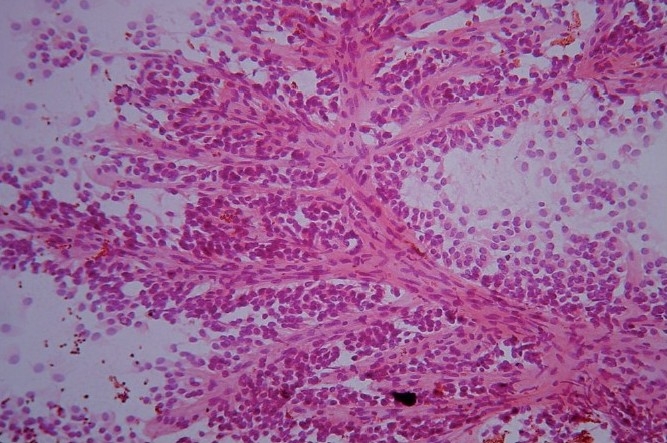
FNAC smear from case of SPT showing tumor cells arranged in papillary pattern and discrete cells (Pap, ×100)

**Figure 2:**
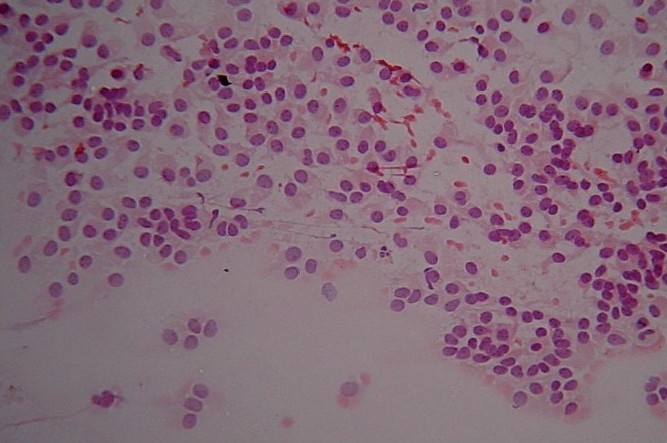
FNAC smear from a case of SPT showing discrete cell (Pap, ×200)

Cytomorphological findings are described in Table 2 and illustrated in microphotographs [Figures 1 and 2].

**Table 2:** Cytomorphological findings

Case	Cellularity	Papillary formation	Discrete cells	Central capillaries	Nuclear grooves/convolutions	BI/Multi nucleation	Back ground	Mitosis
1.	Hyper cellular	+	+ many	+	+	–	Hemorrhagic	–
2.	Moderately cellular	+	+	+	+	+	Hemorrhagic, foamy cells	–
3.	Hyper cellular	+	+ many	+	+	-	Foamy cells, multinucleated giant cells	-
4.	Hyper cellular	+	+ many	+	+	Occasional	Foamy cells, multinucleated giant cells	Rare
5.	Cellular	+	+	+	+	–	Hemorrhagic foamy cells	–
6.	Cellular	+	+	+	+	–	Hemorrhagic	–

In summary, smears were moderate to hypercellular showing papillae formation, discrete cells and occasional pseudorosettes. Pseudorosettes were seen in case no. 1 and 2. The papillae formation was seen in all the cases having delicate vascular cores and covered by two or several layers of cells. All the cases showed discrete tumor cells. The individual tumor cells had round to oval eccentric nuclei with bland nuclear chromatin and moderate amount of pale pink cytoplasm. Case nos. 2 and 3 showed presence of small nucleoli. In all the cases, nuclei showed nuclear grooves and convolution. Binucleation and multinucleation were seen in case nos. 2 and 4. Background was hemorrhagic in all the cases. Case nos. 2–5 showed foamy cells in the background while multinucleated histiocytic giant cells were present in the background in case nos. 3 and 4. Occasional mitosis but not atypical were present in case no. 4.

### Histological findings

Resection specimens were available in four cases and they shared similar macroscopic findings. All were well-circumscribed mass with tan to brown variegated cut surface showing areas of hemorrhage and necrosis. Average maximum tumor diameter was 11 cm (range 6 to 16 cm).

Microscopic findings in all six cases were relatively similar. Histology sections showed sheets and cords of cells arranged around delicate fibrovascular septa. There were marked degenerative changes such as formation of microcysts, hemorrhage, aggregates of foamy cells and cholesterol granulomas. Because of these degenerative changes, cells arranged farthest from blood vessels resulted in a pseudopapillary pattern and pseudorosettes. Tumor cells were small to medium in size with round to ovoid uniform nuclei that often were convoluted and grooved having fine chromatin and inconspicuous nucleoli. Cells had eosinophilic cytoplasm. PAS positive and diastase-resistant hyaline globules were present in all the six cases.

### Immunohistochemistry

Results of immunohistochemistry studies showed strong positivity for Vimentin in all cases. Epithelial markers (CK and EMA) were positive in two of the six cases. Neuroendocrine marker (synaptophysin) was positive in a single case. CD56 and chromogranin were negative in all the cases. These immunostain results were consistent with previously described immunohistochemistry of this lesion.

## Discussion

Solid and pseudopapillary epithelial tumor (SPT) of pancreas is an uncommon tumor with a distinctive clinicopathological profile.[7,8] It is known by a variety of names one of which is “Frantz tumor” as it was first described by Dr. Frantz in 1959.[9] The diagnosis should be suspected in any adolescent or young adult female presented with abdominal mass and radiologically cystic or partially cystic well-circumscribed pancreatic mass. Most SPTs behave in a benign or very low grade malignant fashion and the prognosis after surgical excision is excellent.

Histogenesis of SPTs is unknown due to discrepancies in immunohistochemical and ultrastructural findings;[7] however, the tumor is believed to arise from uncommitted epithelial cells that can show exocrine or endocrine differentiation or both.[10]

A preoperative cytological diagnosis of SPTs is very important due to its implication for management. As these tumors are highly vascular, preoperative accurate diagnosis can avoid complications like hemoperitoneum and intraoperative hemorrhage. This tumor is only locally aggressive, metastasis is rare and so cure from surgical excision is expected in majority of cases. Bondeson *et al*.[11] first described the FNAC diagnosis under ultrasound guidance. Since then a few studies have appeared on cytology findings. The cytomorphology of this tumor is highly characteristic and distinct from those of other cystic or solid tumor of pancreas.[12] The cytologic features in our cases of SPTs were very similar to those described in earlier reports.[2,6,12,13] The highly cellular smears show numerous papillary tissue fragments with slender branching fibrovascular stalks which are characteristic of this tumor. The tumor cells form two or several layers on the fibrovascular core. Pseudorosette formations are also described. In our study, all the cases showed papillary fragments. Pseudorosettes were seen in our two cases.

Apart from these papillary fragments, there were many discrete tumor cells. The individual tumor cells have been described as being monomorphous having round to oval eccentric nuclei, bland nuclear chromatin pattern with small nucleoli and exhibiting longitudinal grooves and convolutions. The cytoplasm being eosinophilic.[6] In our cases, tumor cells showed similar cytomorphology.

In addition to perivascular and papillary fragmentation Jayaram *et al*.[14] considered the presence of intracytoplasmic inclusions as a distinctive feature of this neoplasm which was not seen in any of our case.

Cappellari *et al*.[15] considered the nuclear folds or grooves to be a characteristic of this tumor as seen in all our cases. Binucleation and multinucleation of tumor cells are also described,[12] which were present in two of our cases. Mucinous change in the stalks of papillae, foamy cells and multinucleated giant cells are also mentioned earlier in reports.[2,6,12,13]

Mitosis is not a usual feature of this tumor. In our study occasional mitosis were found one case which was not atypical.

When one encounters cystic lesion in pancreas, pseudocyst constitutes 80% of cystic lesions. Primary cystic neoplasm such as serous cystadenomas, mucinous cystic neoplasms, intraductal papillary mucinous tumor and SPTs constitutes 20%.[16] Rarely do ductal adenocarcinoma and islet cell tumor show secondary cystic change.[6] The various entities in this group occur more frequently in female but differ in age of presentation. Mucinous cystic neoplasm occurs in a wide age range between 20 and 60 years (mean age 47 years). Patient with serous cystadenoma are much older and mean age at presentation varies from 61 to 68 years. Islet cell tumor of pancreas occurs predominantly in adults and show no sex difference while ductal adenocarcinoma occurs predominantly in elderly and show slight male preponderance (M:F; 1.6:1 ratio). In contrast, SPTs occur almost exclusively in adolescent girls and young women. However it may occur occasionally in elder women and rarely in male.[6,17]

Aspirates from pancreatic pseudocyst is sparsely cellular to acellular with variable amount of debris, inflammatory cells, macrophages, occasional columnar cells and metaplastic squamous cells. Raised serum amylase assay and low CEA level confirm benign nature of lesion.

Aspirates from serous cystadenoma which is watery show sparse cellularity with mainly monolayer sheets and occasional papillary fragment of monomorphic cells with vacuolated cytoplasm and bland nuclear chromatin.[17]

The aspirates of mucinous cystic neoplasms are thick and mucoid, which microscopically reveals abundant extracellular mucin and tumor cells. The tumor cells are columnar and either bland and monomorphic or cytologically malignant depending on stage of tumor progression.[18,19]

In case of ductal adenocarcinoma smears show cells arranged in three dimensional clusters, microglandular pattern and occasional papillary fragments with obvious features of malignancy.[6,17,20,21]

Aspirates from islet cell tumors show monomorphic tumor cells arranged dispersely, in loose groups and in pseudorosettes having speckled nuclear chromatin, 1–3 small nucleoli and poorly defined fine granular cytoplasm.[2,17,22]

FNAC diagnosis of SPTs is the characteristic presentation such as young woman with pancreatic mass, well circumscribed with mixed echo density in ultrasonography and characteristic cytomorphology is relatively easy.

## Conclusions

SPT is the only locally aggressive tumor with little to rare incidence of metastasis and therefore a surgical cure can be expected in majority of cases. A preoperative accurate cytological diagnosis is very important so that a surgeon is better informed and better prepared to perform the appropriate surgical procedure, which permits the retention of portion of the uninvolved pancreas (if possible) and avoids the development of subsequent diabetes mellitus.
